# To Approve or not to Approve? A Comparative Analysis of State-Company-Indigenous Community Interactions in Mining in Canada and Sweden

**DOI:** 10.1007/s00267-024-01949-7

**Published:** 2024-03-06

**Authors:** Karin Beland Lindahl, Gary N. Wilson, Christina Allard, Greg Poelzer

**Affiliations:** 1 Political Science, Division of Social Sciences, Luleå University of Technology, Luleå, Sweden; 2 Ájtte – the Swedish Mountain and Sámi Museum, Jokkmokk, Sweden; 3 Department of Political Science, University of Northern British Columbia, Prince George, BC Canada; 4 Law, Division of Social Sciences, Luleå University of Technology, Luleå, Sweden; 5 School of Environment and Sustainability, University of Saskatchewan, Saskatoon, SK Canada

**Keywords:** Interactive governance, Institutions, Mining, Indigenous peoples, Sweden, Canada

## Abstract

This Special Section explores the interplay between Indigenous peoples, industry, and the state in five proposed and active mining projects in Canada and Sweden. The overall aim is to identify factors shaping the quality of Indigenous community-industry-state interactions in mining and mine development. An ambition underlying the research is to develop knowledge to help manage mining related land-use conflicts in Sweden by drawing on Canadian comparisons and experience. This paper synthesizes the comparative research that has been conducted across jurisdictions in three Canadian provinces and Sweden. It focuses on the interplay between the properties of the governance system, the quality of interaction and governance outcomes. We combine institutional and interactive governance theory and use the concept of governability to assess how and why specific outcomes, such as mutually beneficial interaction, collaboration, or opposition, occurred. The analysis suggests there are measures that can be taken by the Swedish Government to improve the governability of mining related issues, by developing alternative, and more effective, avenues to recognize, and protect, Sámi rights and culture, to broaden the scope and increase the legitimacy and transparency of the EIAs, to raise the quality of interaction and consultation, and to develop tools to actively stimulate and support collaboration and partnerships on equal terms. Generally, we argue that Indigenous community responses to mining must be understood within a larger framework of Indigenous self-determination, in particular the communities’ own assessments of their opportunities to achieve their long-term objectives using alternative governing modes and types of interactions.

## Introduction

Resource extraction projects such as mining are often beset by complex social dilemmas and competing interests that complicate decision-making and governance. Mining provides employment and revenues to many local communities but is also associated with negative environmental impacts and conflicts (Bebbington et al., 2008; Hodge, 2014; Conde, 2017; Martinez-Alier et al., 2016; Fjellborg et al., 2022). Many mineral rich areas are located on the traditional territories of Indigenous peoples, and there is a long history of conflicts between Indigenous communities and mining industries (Hilson 2002; Ali, 2009; Hilson and Laing, 2017; Raitio et al., 2020).

Until quite recently, Indigenous peoples in Canada, Sweden and many other countries were largely excluded from any significant role in environmental management or resource development on their traditional lands (O’Faircheallaigh and Corbett, 2005). However, during the last several decades this has started to change. In Canada, the changes are reflected in increased engagement and involvement in the development of impact and benefit agreements (IBAs), environmental assessments (EAs) and other means of Indigenous community collaboration (Collins and Kumral, 2021; Allard and Curran, 2021). Indigenous communities in Canada have become increasingly important governance actors due to a combination of factors, including activism and pressure for Indigenous rights and political, legal and constitutional changes. These developments have empowered Indigenous peoples and established innovative governance arrangements that allow for greater autonomy and collaboration with other levels of government and require those governments to consult with Indigenous peoples (Prno and Slocombe, 2012; Poelzer, 2002; Hunter, 2006; Wilson and Selle, 2019).

In Sweden, much of the country’s mining and mine development has taken place in Sápmi, the traditional territory of the Indigenous Sámi people. Here, Indigenous opposition and disputed rights have caused a standstill in the permitting of new mines during the last decade (Raitio et al., 2020). At the core of this controversy is land use conflicts between resource extraction and reindeer herding, a traditional livelihood of the Sámi (Lawrence and Kløcker Larsen, 2019; Beland Lindahl et al., 2018). Insufficient, or inadequate planning and consultation (Lawrence and Kløcker Larsen, 2017; Sehlin McNeil, 2017; Pölönen et al., 2020) and disputed Indigenous rights in mining legislation and permitting are key issues (Lawrence and Moritz, 2019; Raitio et al., 2020; Tarras-Wahlberg and Southalan, 2022).

This Special Section explores the interplay between Indigenous peoples, industry, and government in five proposed and active mining projects in Canada and Sweden: Prosperity/New Prosperity (British Columbia, BC), MacArthur River/Key Lake (Saskatchewan) and the Diavik Mine (Northwest Territories, NWT) in Canada, and Kallak/Gállok (Jokkmokk municipality) and Aitik (Gällivare municipality) in Sweden. The research has been conducted as part of a project^1^ that compares company-Indigenous community interactions across a number of jurisdictions in Canada and Sweden, and aims to develop knowledge and tools to manage mining related land-use conflicts in Sweden involving Indigenous communities (Sámi Reindeer Herding communities, RHCs) by drawing on Canadian comparisons and experiences.

Much of the research on company-community interactions in mining is framed around the concept Social License to Operate (SLO). In short, SLO refers to the approval and ongoing support of resource projects by rightsholders and stakeholders that are directly impacted by these projects (Prno, 2013; Prno and Slocombe, 2012; Thomson and Boutilier 2011). The SLO literature has been extensively reviewed in other publications (e.g., Lesser et al., 2020) which show how the concept has gained traction while being criticized by practitioners and scholars (Boutilier, 2020; Hitch et al., 2020; Owen and Kemp, 2013). Prno and Slocombe (2012) place SLO within a larger governance context arguing that other stakeholders, markets, contextual variables – and institutions – interact to produce outcomes (see Pierre and Peters, 1998; Kooiman, 2003). Accordingly, previous SLO research has highlighted the importance of the governance context and the existence of different multi-sectoral and multi-scalar governance structures (Prno, 2013; Prno and Slocombe, 2012; Boutilier, 2020; Lesser et al., 2020). However, while the Prno and Slocombe (2012) model helps place company-community interactions, and SLO, in a governance context, it does not provide much guidance about to how to analyse the interactions shaping the quality of state-company-community relations. Haslam (2021) underscores the role of companies and their social practices, Boutilier (2020) stresses the need to further analyze actors’ power relations, but little attention has been paid to the role of the state and its institutions.

The bulk of the SLO literature does not explicitly address Indigenous people and their specific challenges. Some studies suggest that SLO related activities may change power dynamics in positive ways (e.g., Ritter 2009), but others question the long term benefits (e.g., Collins and Kumral, 2021). The broader literature on environmental management addresses Indigenous participation in greater depth, including the practice to negotiate agreements between industry and Indigenous communities. O’Faircheallaigh (2021) suggests that the combination of legal recognition, political and cultural mobilization, and avenues for community participation have created the political capacity to achieve positive outcomes but calls for research that can explain the reasons for observed differences in outcomes. Similarly, Ali (2009) argues that scientific criteria alone cannot explain the emergence of Indigenous resistance and proposes that the primary issue at stake for contemporary Indigenous communities in North America is a reassertion of their sovereignty.

The aim of this paper is to identify factors shaping the quality of state-company-Indigenous community relations by comparing governing interactions and outcomes in mining and mine establishment across Canadian and Swedish jurisdictions. We combine institutional and interactive governance theory and use the concept of governability (Kooiman, 2003; Jentoft and Chuenpagdee, 2015a) to assess how and why specific outcomes, such as mutually beneficial interaction or collaboration, occur.

## Theoretical Framework

**Fig. 1:**
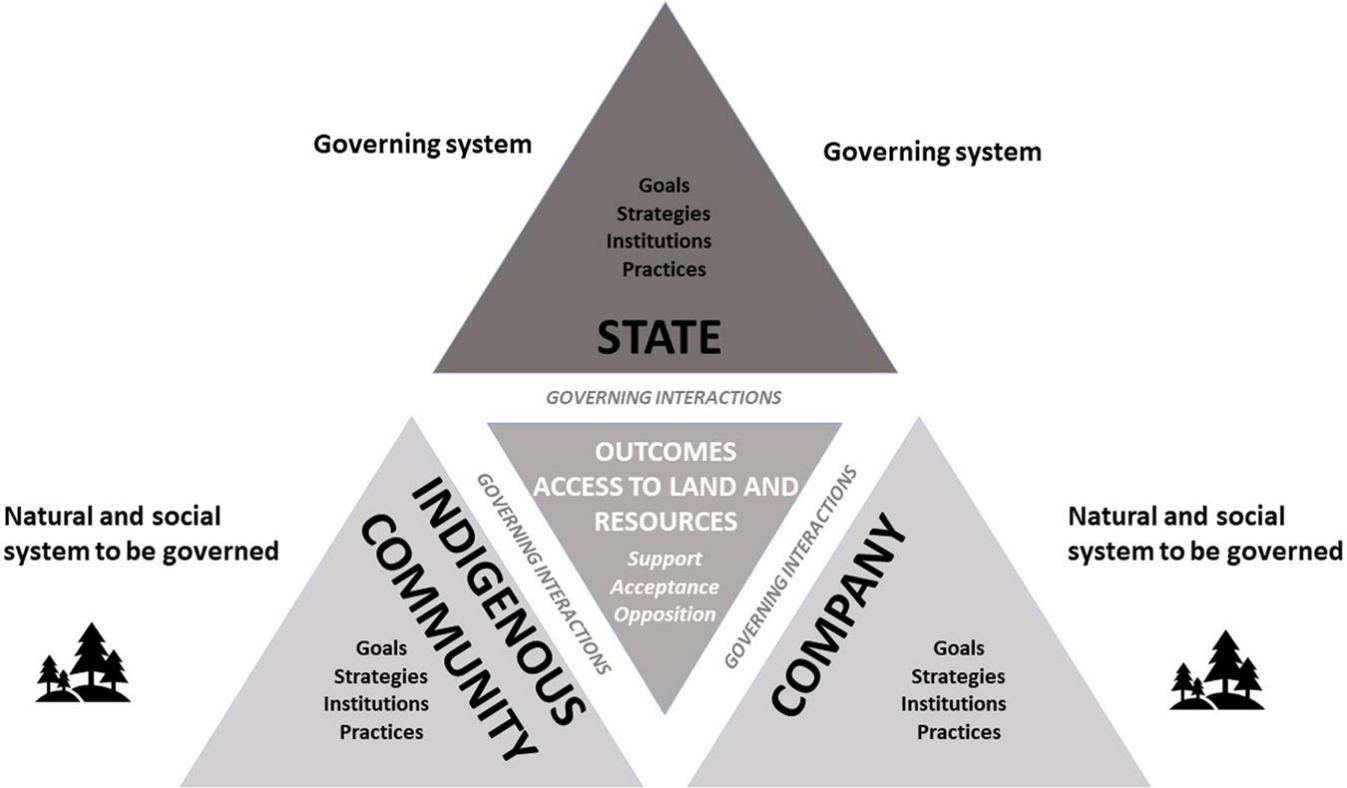
Analytical framework developed from Prno and Slocombe (2012) and Jentoft and Chuenpagdee (2015a, 2015b)

This study draws on institutional and governance theory and uses Prno and Slocombe’s (2012) model of state, society, and market interaction in mining activities as a point of departure. However, interactive governance theory offers additional tools to assess the performance of different *governance systems* considering the diversity, complexity, dynamics and scale that characterize these systems (Kooiman, 2003; Jentoft and Chuenpagdee, 2015a). A governance system includes (i) the natural and social *system-to-be-governed* (company and Indigenous community); (ii) the *governing system* (the state) and (iii) the *governing interactions* that occur between these entities (see Fig. 1). Jentoft and Chuenpagdee (2015a) introduce the concept of *governability* (see Kooiman, 2003) to assess the overall quality of governance. First, this concept addresses the characteristics of the system-to-be-governed that may contribute to making the system more or less governable. Second, it assesses the capacity of the governing system to handle these characteristics and address societal concerns. How the governing system performs its functions, whether it corresponds and responds to the system-to-be-governed, and how these two systems interact with each other are key aspects of governability. Further, as Fig. 1 demonstrates, governability analysis helps us to understand whether governing instruments (e.g., state and corporate strategies, institutions and practices) suit their purposes, and whether governance processes and *outcomes* are aligned with universal meta-order values and standards; for example principles of democracy and agreements on Indigenous rights (Jentoft and Chuenpagdee, 2015a; 2015b).

Institutions, here understood as the formal and informal “rules of the game” (North, 1990), constitute an important part of the governing system (see Pierre and Peters, 1998; Kooiman, 2003; Young et al., 2008). Institutions may be enabling (incentive based), constraining (regulation based), formal (law-based) and informal (customs-based, Mehta et al., 2001). Accordingly, the institutional context governing a particular situation structures interaction by presenting the actors with a particular distribution of opportunities and constraints; invariably, this distribution favors particular understandings, actors and actions while excluding, marginalizing and guarding against others (Jessop, 2001; Hay, 2002). Concurrently, different types of interaction and action may alter, or give rise to new, institutions (Mahoney and Thelen, 2010; Capoccia and Keleman, 2007). Governability is not a static phenomenon but changes with the interactions that occur within and between the governing system and the system-to-be-governed.

At the forefront of interactive governance is the issue of governing interactions and the significance of the nature, type and quality of these interactions. Jentoft and Chuenpagdee (2015a) differentiate between three modes of interaction: *self-governance, co-governance* and *hierarchical governance*. Self-governance refers to systems, or parts of systems, that have the ability to govern themselves without much external interference or support. In a co-governance arrangement, the government (at any level) acts as a constructive partner in the governance of a resource or area. This may take the form of co-management or other kinds of partnerships or arrangements facilitating participation. Finally, hierarchical governance refers to any kind of governance arrangement that operates according to a top-down order (Jentoft and Chuenpagdee, 2015a). It typically involves states but can also be found within local governments, communities and corporations. In the context of Indigenous resistance and mining, Ali (2009) stresses two elements that shape patterns of interaction: *player linkage* and *issue linkage*. The former refers to the nature of alliance formation between different actors and highlights the importance of different stakeholders’ varying opportunity costs and alternatives to negotiated agreements. Issue linkages address how the linkage of issues can help or hinder the attainment of the ultimate objectives for each actor. Issue linkages can be synergistic or antagonistic depending on whether they increase or decrease the zone of agreement. They can also be reciprocal or competitive. Competitive linkages occur when agreement in one context precludes agreement, or attainment of objectives, in other related contexts (Ali 2009).

Drawing on Chuenpagdee and Jentoft (2013), we developed a framework (see Fig. 1) to guide a systematic comparison of mineral governance on Indigenous territories in different Canadian and Swedish sub-national jurisdictions. The first step explores the natural system to be governed (land and natural resources); the governing system (state); and the social system to be governed (company and community). The system properties under examination include geography, land use and mineral resources; governing states and institutions that regulate resource use and interactions; and the actors’ goals, strategies and practices. The second step focuses on analyzing governing interactions, including player and issue linkages, while the third looks at outcomes and governing performance. Evaluating governing interactions is about assessing the significance of the type and quality of the interactions and what difference the various governance modes (co-, self-, hierarchical) and linkages (player and issue) make to the interactions, particularly in terms of quality. At its core, therefore, governing performance is about the capacity of the governing system to address societal concerns, in this case about planned or on-going mining activities. Governing performance can also be assessed in terms of outcomes, such as the question of who can access the natural resources and the level of community opposition, acceptance or support of mineral extraction. Figure 1 illustrates the interactions between the different parts of the governance system (state, community and company) and the outcomes by using a symmetric triangle. However, as highlighted by Ali (2009) the bargaining power of state, companies and Indigenous communities may be highly unequal and is subject to empirical investigation.

Reflecting on the framework above, our overarching question is: *How do system properties, governing interactions, and governing performance interact to produce outcomes?* Specifically, we ask the following questions (RQs):

RQ 1: What is the significance of system properties and how do they vary between the cases?

RQ 2: What is the significance of modes, linkages and type/quality of governing interactions and how do they vary between the cases?

RQ 3: How do the cases vary in terms of governing performance and outcomes?

## Methodological Approach

Canada and Sweden are two highly regulated countries with large mining industries operating on Indigenous territories. On the one hand, the two countries are broadly similar in the sense that they are both western democracies with economically important mining industries facing similar discussions about SLO and the role of Indigenous peoples in decisions around resource development. On the other hand, Canada is approximately 20 times larger than Sweden and has a diversity of institutional and governance arrangements (federal/provincial/territorial as well as Indigenous) that are significantly different from those of Sweden and vary between different jurisdictions and communities. Moreover, Canada offers many examples of innovative collaborative arrangements by which Indigenous communities, companies, and government share responsibility for environmental stewardship and community development. These differences provide unique opportunities to study the interplay between the properties of the governance systems, interactions and governing performance and outcomes. Three cases in Canada and two cases in Sweden were selected for study (see Table 1). More specifically, the cases were chosen to illustrate proposed as well as operating mines in both countries, a range of commodities, and a variety of institutional conditions (treaties, agreements and governance arrangements) in the Canadian cases. The field work and data collection took place during 2019 and 2020.

**Table 1:** Case Studies

Cases	Prosperity	McArthur River/Key Lake		Diavik	Kallak/ Gállok	Aitik
Location	British Columbia Canada	Saskatchewan Canada		Northwest Territories Canada	Jokkmokk Sweden	Gällivare Sweden
Commodity	Gold/Copper	Uranium		Diamond	Iron	Copper
Project stage	Proposed mine	Licensed to operate; production restart awaiting improved markets		Operating mine	Proposed mine	Operating mine Expansion
Owners	Taseko	Cameco and Orano		Diavik Diamond Mines	Jokkmokk Iron Mines AB	Boliden
Indigenous community	Tshilhqot’in Nation	English River First Nation and others		Tlicho Government	Jåhkågaska tjiellde and others	Gällivare Skogssameby
Institutional characteristics	Stronger Indigenous rights				Weak Indigenous rights	Weak Indigenous rights
	No treaty but strong aboriginal rights		Historical Treaties Long tradition of IBAs	Modern Treaties Multi-level governance		

A common research guide was developed to streamline the research process and ensure consistency between the work conducted by the researchers. The research questions (RQ1-3) guided the empirical investigation of all cases. The methods consisted of document analysis, including legal and other official documents, websites and other texts produced by the actors themselves, and media sources, as well as qualitative interviews with representatives of key Indigenous community and corporate actors. A jointly developed interview guide was applied, and the semi-structured interviews were recorded and transcribed. However, due to restrictions caused by the Covid-19 pandemic, the original data collection plan had to be adjusted. Several of the planned interviews on the Canadian side had to be replaced by more in-depth document analysis and reviews of existing scientific studies. Only one interview could be conducted in the Diavik case (with a former representative of the T’licho Investment Corporation) and one in the Prosperity case (with a representative from the Indigenous community). Four formal interviews (two with Government of Saskatchewan officials and two with the Indigenous owned Des Ned’he Development) were conducted in the McArthur River/Key Lake case. In this case, the researchers were also able to communicate freely with representatives of the mining company (Cameco Corporation). Altogether nine interviews (three with company representatives, four with the involved Sámi RHCs and two with their legal representatives) were conducted in the Swedish cases. This comparative analysis also drew on the already completed articles in this Special Section exploring the individual cases and their institutional context (Allard and Curran, 2021; Wilson and Allard, 2023; MacPhail et al., 2022; Jackson et al., 2023; Poelzer et al., 2023; Poelzer, 2023). All empirical information was systematically compiled, analyzed thematically in relation to RQ1-3 and compared to identify important similarities and differences between Canada and Sweden generally and the individual Canadian and Swedish cases specifically. More nuance and depth regarding corporate and Indigenous goals and strategies in the Canadian cases could probably have been gained with additional interviews. However, all three Canadian cases were already well documented, and the research team had prior contextual knowledge about the Diavik and the McArthur River/Key Lake cases.

## Results and Comparative Analysis

Below is a summary of the main results, including the comparative analysis of the regulatory frameworks in Canada and Sweden and the cases (see appendix 1 for a more comprehensive description).

### System Properties

RQ 1 explored the significance of system properties (geography, land use and mineral resources; governing states and institutions; corporate goals, strategies and practices; and Indigenous goals, strategies and practices). Table 2 summarizes the main differences and similarities of these properties.

**Table 2:** Main similarities and differences of the system properties characterizing the governance systems of the selected cases

Case(s)	System properties					
	Geography and land use/natural resources management	Governing states and institutions		Corporate goals, strategies and practices	Indigenous goals, strategies and practices	
Prosperity, BC, Canada	Land use competition and conflicts not resolved. Controversial location of proposed mine.	Federal system with overlapping federal and provincial laws. Common law system with greater litigation opportunities Stronger constitutional recognition of Indigenous rights A State duty to consult. Government to government type relationships Legal and political objective of reconciliation	Inclusive EA conducted by independent EA assessment authority. Separate provincial and federal EA. Several decisions appealed.	Existing CSR policies not demonstrated in practice. Failure to establish trust and communication and reach stage when collaboration could be realized.	To maintain or develop sustainable livelihoods, revitalize cultures and develop opportunities for future generations. Increased self-determination and/or respect for Indigenous rights, traditional knowledge, and ways of life.	Opposition to protect traditional livelihoods and sacred site, and to advance goal of self-governance and aboriginal title to its traditional territory
McArthur Key Lake, Saskatchewan, Canada	Land use competition and conflicts addressed through company- community collaboration.		Surface Lease Agreements. Historical Treaties Variety of well-developed private agreements.	To establish trust, business partnerships and collaboration. Well-developed IBAs on benefits sharing, environmental stewardship, relationship building and collaboration.		Collaboration and partnerships with companies (and state) to ensure benefits, influence and control.
Diavik, NWT, Canada	Less intense land use competition handled by co-management.		Co-Management Boards. Indigenous self-governance Variety of well-developed agreements, including private. Modern Treaties			
Kallak/Gállok, Jokkmokk, Sweden	Intense land use competition and cumulative impacts caused by resource extraction not resolved. Controversial location of proposed mine.	Unitary state legislation. Weaker constitutional recognition of Sami rights No State duty to consult Sami applied at the time of investigation. Sami reindeer herding treated as an industry and as any other Swedish public interest. Narrow scope of EIA.		Efforts to restore relations after bad start. Failure to establish trust and communication with affected Sami		Opposition to protect reindeer herding and gain respect for Sami rights.
Aitik, Gällivare, Sweden	Intense land use competition and cumulative impacts by resource extraction, including ongoing mining, not resolved. Controversial location of proposed mine expansion.			Consultation and initiation of some collaborative activities. Private agreements on mitigation and compensation.		Consultations, negotiation, (some collaboration) and opposition (Liikavaara) to protect reindeer herding.

#### Geography, Land use and Mineral Resources

Some important aspects of the systems to be governed are their geographical conditions, land use values and practices, and the type of targeted mineral resources. Saskatchewan, BC, NWT and northern Sweden are regions with long traditions of mining, and the targeted commodities include copper and gold (Prosperity and Aitik), iron (Kallak/Gállok), uranium (McArthur River/Key Lake) and diamonds (Diavik). All cases are located in sparsely populated and historically resource dependent regions where forestry, mining and traditional economic activities such as hunting, fishing and trapping are important (see Beland Lindahl et al., 2018; Jackson et al., 2023; Wilson and Allard, 2023; Poelzer et al., 2023). Land use competition or conflicts are prominent issues in both countries; in Sweden, Sámi RHCs depend on reindeer herding which requires extensive territories and is under pressure by cumulative land use impacts (Lawrence and Kløcker Larsen, 2019), and in Canada, Indigenous communities rely on traditional hunting, trapping and fishing which also is sensitive to the impacts of resource extraction. Therefore, the geographical context, the extent of competing land use values/practices and the location of mines play important roles in determining outcomes.

In the cases with open conflicts (Prosperity and Kallak/Gállok), or significant Indigenous dissatisfaction (Aitik), the proposed mines were located in less sparsely populated areas, and/or in places with documented land use conflicts, and in proximity to villages and population centers with non-Indigenous majority populations, or in culturally or environmentally sensitive locations. The proposed, or expanding, mines in the Prosperity, Kallak/Gállok and Aitik cases threatened sacred sites, areas identified as being of “national interest” and strategic passages for reindeer herding in ways that were perceived as non-acceptable (MacPhail et al., 2022; Poelzer, 2023). In the Diavik case, where collaboration and partnerships evolved, land use competition was less intense, and the location of the mine was less controversial in terms of its position relative to communities.^2^ Northern Saskatchewan, where the McArthur River/Key Lake case is situated, was likewise very sparsely populated and the mines are located far from the population centers. Nevertheless, uranium extraction is associated with significant environmental risks and, as a result, environmental impacts have become important issues. However, in this case it was possible to resolve land use and environmental issues through communication and mutually appreciated collaboration between the company and the Indigenous community (Jackson et al., 2023; Poelzer et al., 2023).

In response to RQ1, the analysis suggests that the geographical context, the extent of competing land use values/practices and the location of mines play important roles in shaping interactions and outcomes in both countries; however, combining traditional Sámi reindeer herding and large-scale mining, as is the case in Sweden, constitutes a significant governance challenge.

#### Governing States and Institutions

Another key aspect of the governance system in both countries is the state and its institutions. Although Canada and Sweden are both liberal democracies, their institutional contexts differ (see Table 2 and App. 1). Our three Canadian cases are situated in two provinces (BC and Saskatchewan) and in the NWT. All three jurisdictions have their own distinct laws and regulatory systems, alongside federal laws and regulatory systems. The jurisdictional and legal landscape is complex and makes a comprehensive overview of mineral extraction in the Canadian and Swedish cases impossible. However, key features are highlighted below.

##### Constitutional Protection of Indigenous Rights

Section 35(1) of Canada’s *Constitution Act, 1982* includes an explicit protection of Indigenous rights, which has been interpreted and developed by the Supreme Court of Canada (SCC). Although the Constitution Act refers to aboriginal peoples, today the most commonly accepted term is Indigenous peoples, which include First Nations (Indians), Inuit and Métis.^3^ Sweden has one recognized Indigenous people, the Sámi. The Swedish constitution, the *Instrument of Government, 1974*, allow some degree of protection of Sámi rights and culture, but not to the same extent as in Canada (Allard, 2015, p 50, 54).

One consequence of the constitutional protection of Canada’s Indigenous peoples is the state duty to consult, developed over time by a series of Supreme Court cases (Newman, 2014). It places a legal requirement upon the Crown (federal and provincial governments) to consult when they are making a decision that could affect Indigenous rights, and an obligation to accommodate the potentially affected Indigenous communities (Government of Canada, 2021). Whether consultation has been adequate or not is often a source of controversy and may be litigated before the courts. The McArthur/Key Lake case demonstrated that respect for Indigenous rights and the involvement of Indigenous peoples in decision-making about mining projects have been operating principles of governments and companies for several decades. Sweden has enacted new state legislation in 2022 imposing a duty for the government and state agencies to consult with Sámi in matters of particular significance to them, including mine development. However, this legislation predates the current analysis of the Swedish cases, which only included *corporate* consultations with Sámi as part of environmental impact assessments.

The Canadian Constitution also protects “treaty rights”.^4^ In both Pre- and Post-Confederation Canada, the Crown used historic treaties to negotiate terms with Indigenous in order to secure land title and foster peaceful relations; the era of historic treaties ended in the 1920s. Since the 1970s, the Government of Canada has pursued modern treaties, which are more comprehensive in nature and reflect the evolution of jurisprudence on Indigenous rights. Nevertheless, the respective rights conveyed in each treaty - historic and modern - are enshrined in the Constitution Act (Government of Canada, 2023). A large proportion of Canada is under historical treaties, except for most of BC (Government of Canada, 2013). Saskatchewan, where the McArthur/Key Lake case is situated, includes land covered by several historical treaties (ibid). Such treaties have played a significant role in the increasing legal recognition of Indigenous rights in Canada. Today, there are multiple strategies being employed to recognize and advance Indigenous rights, including negotiated modern treaties in areas not covered by historical treaties. While the Tlicho Government in the NWT has signed modern treaties, the Tsilhqot’in Nation in BC is unique in that it does not have a treaty with the Crown but has secured aboriginal title (ownership) to a portion of its territory through litigation.^5^ Neither the Swedish Crown nor contemporary governments have made any such treaties with the Sámi.

##### Reconciliation and Partnerships

The Government of Canada has since long engaged in an active reconciliation process with Indigenous peoples (Government of Canada, 2023), reflecting the fact that reconciliation is a legal and political objective. Accordingly, Canada has promoted flexible governance mechanisms and partnerships with corporate actors to help ensure sustainability and protect the rights and interests of Indigenous peoples (Long, 2019; Jackson et al., 2023). This approach has facilitated the development of various kinds of industry-state agreements, such as Mine Surface Lease Agreements (MSLA) in Saskatchewan and industry-state-community agreements in the Diavik case (Poelzer et al., 2023). In short, MSLAs provide long-term rental of Crown land for mine operations, and, at the same time, place obligations on the mine operator to undertake their best efforts to maximize benefits for local communities.^6^

By contrast, the Swedish state does not embrace the principle of reconciliation with the Sámi people, or promote partnerships in the same way, although there have been some steps taken to improve Sámi self-determination (Mörkenstam et al., 2016; Lawrence and Mörkenstam, 2016). While the Sámi RHCs are autonomous legal entities, they only organize a minority of Swedish Sámi (those who practice reindeer herding), and the Indigenous rights of the RHCs are not recognized in legislation but viewed as a public interest to be balanced against other public interests (Raitio et al., 2020).

##### Environmental Assessments

An important aspect of mine approval processes in both Canada and Sweden is environmental assessments (EA, Allard and Curran, 2021). EA requirements for mining operations vary across Canada, depending on the jurisdiction in which the project is located, the size of the proposed operation, and the potential for adverse effects. Several First Nations governments have their own authority to undertake EA processes because of modern treaties. This was the case when Diavik established its mines in the NWT. And, in BC new EA legislation allows First Nations to undertake Indigenous-led assessments, by which First Nations can be rewarded extra economic compensation for such work. A complicating factor in the context of large mining projects in Canada is overlapping jurisdictions between federal and provincial governments. Normally, they collaborate on the EAs, but on certain occasions, for example in the Prosperity case, two EAs were carried out for the same project. In this case, the federal EA process involved more extensive community hearings over a longer time-period and did not approve the EA whereas the provincial EA process was less consultative and came to the opposite conclusion.

The Swedish environmental impact assessment process (EIA) differs from the typical Canadian EA because it is tightly connected to the permit process for specific mine projects (Pölönen et al., 2020). The EIA is the sole responsibility of the proponent, both in terms of collecting scientific knowledge and carrying out (corporate) consultations with affected parties including Sámi RHCs. The new legislation on the duty to consult Sámi will modify this procedure, but to what extent remains to be seen. The Swedish EIA typically addresses *environmental* impacts only and has a narrower scope than the Canadian EA which also addresses social and cultural impacts, and long-term effects on Indigenous rights. Compared to the Swedish, the Canadian EA is also more autonomous; in BC, for instance, EAs are organized and supervised by the Environmental Assessment Office, an agency under the provincial government (Allard and Curran, 2021).

In response to RQ1, about the significance of governing states and institutions, we can conclude that Indigenous communities in Canada enjoy a broader palette of opportunities to advocate their Indigenous rights compared to the Swedish Sámi. The combination of stronger constitutional rights in Canada, active reconciliation efforts and state-led initiatives such as the MSLAs, enabled Indigenous communities to engage directly with governments, companies and/or litigate decisions. Over time, the Crown’s willingness to bestow a degree of autonomy to Indigenous governments has leveraged their standing and supported Indigenous involvement, participation and, sometimes, collaboration in state and corporate decision-making processes.

#### Corporate Goals, Strategies and Practices

Another aspect of the systems to be governed are the companies and their goals, strategies and practices. Differences exist between corporate cultures in Canada and Sweden (Poelzer, 2023). Although not expressed in practice in all investigated cases, Canadian companies generally operate with more extensive corporate policies, company-community engagement protocols, and build more comprehensive private agreements compared to companies in Sweden. In Europe, there is typically a greater degree of trust in government bodies and their roles in safeguarding the environment and social benefits (Lesser et al., 2021).

Different corporate approaches are also reflective of the position of Indigenous peoples and their constitutionally protected rights, which are generally stronger in Canada (see above). MSLAs and tripartite industry-state-community agreements were part of the Canadian Government’s efforts to protect Indigenous rights (Long, 2019; Jackson et al., 2023). For example, the MSLA related to the McArthur/Key Lake project paved the way for a variety of other agreements which boosted company-community cooperation. Indigenous communities in Canada, particularly those with strong aboriginal and treaty rights, have their own governments and land bases which make them “state-like” rather than simply “community-like” actors. This has leveraged their standing in negotiations with corporate actors and facilitated partnerships and collaboration, as reflected in both the Diavik and McArthur/Key Lake cases. Reflecting these differences, the use of privately negotiated Impact Benefit Agreements (IBAs) between companies and Indigenous or local communities, is generally more developed in Canada (Poelzer et al., 2023). In Sweden, questions regarding compensation, mitigation, environmental monitoring, etc. are typically handled as integrated parts of the approval processes under state legislation.

In spite of these differences, collaboration and private agreements between companies and Indigenous communities occurred in both countries. In the Diavik and McArthur/Key Lake cases, IBAs were used to ensure mutual benefits and include provisions for benefits sharing, environmental stewardship, ongoing relationship building and collaboration (Jackson et al., 2023; Poelzer et al., 2023). The approaches applied in these cases were formalized and institutionalized to a higher degree than the mitigation and compensation-focused agreements used in the Swedish Aitik case (Poelzer, 2023). A shift towards long-term partnerships and mutual learning in the Aitik case has begun with research agreements, but the Sámi RHC maintains that existing agreements are limited in scope and calls for more equal negotiations and shared ownership of projects (Poelzer, 2023).

The most advanced examples of corporate-community engagement and partnerships were found in the Canadian Diavik and McArthur River/Key Lake cases. Here, collaboration evolved under particular challenges that also may have opened up opportunities. The McArthur River/Key Lake case involved the mining of uranium, a strictly regulated and publicly contested commodity. In this case, the MSLA agreed to by the corporation and the provincial government provided the motivation for the development and implementation of the company’s (Cameco) progressive CSR practices (Jackson et al., 2023). In the Diavik case, the company wanted government permission, and local support, to extract diamonds during a time when power was being transferred to Indigenous nations in the NWT (Poelzer et al., 2023).

In the conflictual cases (Prosperity and Kallak/Gállok), the interactions between the companies and Indigenous communities never developed to a stage where collaboration could be established and existing CSR policies realized. The initial contacts failed to establish trust or incentives for further engagement, and interactions with the companies were not perceived as meaningful by the Indigenous parties (MacPhail et al., 2022). From their point of view, the proposed locations were not suitable for mine development, consultation was not adequate, and the preconditions for further negotiations and collaboration were, therefore, not in place (ibid).

In response to RQ1, we can conclude that *corporate goals, strategies and best practices* vary across the cases but are generally more extensive and developed in the Canadian cases, particularly those involving IBAs. Corporate goals and strategies generally appear to be important system properties, as demonstrated in the cases were collaboration, partnerships and support developed. However, the conflict cases illustrate how inadequate, inappropriate, or insufficient, corporate engagement strategies may contribute to a lack of trust, communication and engagement.

#### Indigenous Communities’ Goals, Strategies and Practices

Critical to the development of the different cases are the goals and strategies of the affected Indigenous communities. All communities express a desire to maintain or develop sustainable livelihoods, revitalize cultures and ensure opportunities for generations to come (McPhail et al., 2022; Poelzer et al., 2023; Poelzer, 2023). However, one general difference between the Canadian and Swedish cases is the Swedish Sámi RHCs’ overarching aspiration to maintain traditional reindeer herding. Hence, mining in the Swedish Sámi context is primarily associated with the direct and indirect loss and degradation of reindeer pastures, the obstruction of migration routes, activities that disturb the reindeer, and associated economic and socio-cultural impacts (Kløcker Larsen et al., 2022). From the outset, issue linkages were antagonistic and competitive. The Canadian cases displayed a more varied picture where issue linkages to a higher degree depended on the specific goals and strategies of the Indigenous communities in question. A more developed and articulated pursuit of Indigenous self-determination and self-governance in Canada also appeared to shape Indigenous strategies in relation to mining (cf. Ali, 2009).

In the two Canadian cases where collaboration and partnerships evolved (McArthur River/Key Lake and Diavik), the negative effects on the environment and traditional livelihoods were resolved through negotiation in settings that strengthened and reinforced the Indigenous communities’ sense of respect, sovereignty and control. Player linkages, i.e., relations and alliance formation, between the Indigenous communities, the companies, and other governing actors developed, mining primarily came to be associated with positive socio-economic impacts and the issue linkages were largely seen as synergistic and reciprocal.

In the Prosperity case, the environmental and cultural impacts of a mine in the vicinity of Teztan Biny/Fish Lake, an area valued for hunting, fishing, trapping and for ceremonial and spiritual purposes, were deemed unacceptable by the Indigenous community (TNG). TNG advanced a vision for land use based on the values that activities must not unduly harm the land and water (McPhail et al., 2022, p. 12). Moreover, consultation and negotiation with the company was never established, differences between the provincial and federal governments complicated the mine approval process, and the EA process was not deemed capable of adequately addressing aboriginal title or rights (McPhail et al., 2022). Issue linkages were antagonistic and competitive and player linkages were weak. However, TNG did not unconditionally dismiss the potential positive economic benefits of mining on their territories (Tŝilhqot’in National Government, 2023).

While the issue linkages in both Swedish cases were antagonistic and competitive, the nature of the issues differed. The proposed Kallak/Gállok mine does not have all required permits, no mining currently takes place in Jokkmokk municipality, and the affected Sámi RHCs are mobilizing to stop this mine project (Beland Lindahl et al., 2018; MacPhail et al., 2022). The existing Aitik mine has been in operation since the late 1960’s and the impacts, including the accumulated loss of pastureland have created an extremely challenging situation for reindeer herding (Lawrence and Klocker Larsen, 2019). The RHC opposes the proposed mine expansion in Liikavaara and maintains that they never accepted or approved mining on their territories but are nevertheless forced to adapt and interact with the company and the majority of the local population who depend on the mines (Poelzer, 2023). Consequently, player linkage is very different in the two cases. While the RHCs in the Kallak/Gállok case have built alliances with environmental-NGOs and human rights organizations who support their goal to stop the mine, the Gällivare Sámi RHC (Aitik) remains relatively isolated in their negotiations with the company and other local actors who generally promote, or support, mining (Poelzer, 2023; Beland Lindahl et al., 2018). For the Sámi RHCs, the mining projects primarily involve issues about the future possibilities for maintaining and developing Sámi livelihoods and culture associated with reindeer herding. However, especially in the Kallak/Gállok case, it is also a question of recognizing land rights, in other words about gaining more authority and control over land use in the longer term (MacPhail et al., 2022).

In response to RQ1, Indigenous communities’ goals and strategies, appear to be extremely important system properties that may explain different outcomes. Antagonistic and competitive issue linkages due to land use competition and clashing Indigenous and corporate values in the Prosperity, Kallak/Gállok and Aitik cases promoted discord and opposition to the mining projects. However, differences in player linkage can explain why confrontational strategies evolved in the Prosperity and Kallak/Gállok case, but not in Aitik. In the McArthur/Key Lake and Diavik cases, issue linkages were, or had become, predominantly synergistic and reciprocal. Hence, player linkages between the Indigenous communities, the companies and other governing actors, grew strong, and collaboration and support developed.

### Governing Interactions

The second research question explored governing interactions, more specifically the modes (hierarchical-/co-/self-), linkages (player and issue) and type/quality of governing interactions. As shown in Table 3, three “types” of interactions were identified in the investigated cases, and the quality of interactions varied across the countries and cases. Well-developed and mutually appreciated collaboration and partnerships between communities, industry, and sometimes government (McArthur River/Key Lake and Diavik) were only found in some of the Canadian cases. So too were formalized co-management organizations (Diavik). Poorly functioning interactions and open conflicts between Indigenous communities, companies and governmental bodies were seen in both Canadian and Swedish cases (Prosperity and Kallak/Gállok).

**Table 3:** Governing Interactions and outcomes in the five cases

Case(s)	Type/quality of interaction and issue linkage	Modes of interaction and player linkage	Outcomes
McArthur Key Lake, Saskatchewan, Canada	Mutually appreciated collaboration and partnerships. Synergistic and reciprocal issue linkages.	*State-community:* hierarchical *State-company:* hierarchical and co-governance *Company-community:* privatized self- and co-governance through MSLA framework Strong *player linkages* between community and company	Project broadly supported; licensed to operate; production restart contingent on improved markets
Diavik, NWT, Canada		*State-community:* hierarchical and co-governance *State-company:* hierarchical and co-governance *Company-community:* privatized self- and co-governance Strong *player linkages* between community, company and government	Project broadly supported; licensed to operate; ongoing mining
Aitik, Gällivare, Sweden	Dialogue and collaboration without Indigenous consent. Antagonistic and competitive issue linkages.	*State-community:* hierarchical *State-company:* hierarchical but collaborative *Company-community*: privatized self-governance but hierarchical Weak *player linkages* between community and other actors	Aitik project accepted by compliance; licensed to operate; ongoing mining
			Expansion in Liikavaara opposed; mining permit approved by Mining Inspectorate and environmental permit approved by the Land and Environmental Court
Prosperity, BC, Canada	No functioning dialogue and high conflict level. Antagonistic and competitive issue linkages.	*State-community:* hierarchical *State-company:* hierarchical *Company-community*: non existing Weak *player linkages* between community, company and government Strong player linkages with E-NGOs and human rights organizations	Project opposed; mining permit rejected by federal Government
Kallak/Gállok, Jokkmokk, Sweden			Project opposed; mining permit approved by Government.

The McArthur River/Key Lake and Diavik cases revealed well developed collaboration and partnerships which appear to be highly valued by the Indigenous communities and corporate actors. The quality of interaction was generally assessed as high and no major disputes, critical interventions, or legal litigation were formally documented (Jackson et al., 2023; Poelzer et al., 2023). These cases underscored the importance of community involvement and recognition of rights and illustrate how legal rights and a governance role for Indigenous communities helped prevent conflicts (Poelzer et al., 2023). Both cases highlighted the important governing roles of the government: to institute enabling legal and governance conditions using a hierarchical mode of governance and, when appropriate, remain passive and allow the Indigenous and corporate actors to take more active roles in the management of the resources (Poelzer et al., 2023). In the McArthur/Key Lake case, the MSLA initiated by the provincial government and agreed to by the corporation provided the motivation for the development and implementation of responsible CSR and community engagement practices (Jackson et al., 2023). The Diavik mine was developed in tandem with Mackenzie Valley Resource Management Act (MVRMA), an initiative that changed the traditional hierarchical mode of state governance to a system of shared decision-making power based on co-management boards and led to the evolution of a self-governance agreement between the Government of NWT, the Government of Canada and the Tlicho First Nation. These processes were marked by the involvement of Indigenous corporate actors (Indigenous led businesses), different forms of partnerships or formalized co-management structures that institutionalized a devolution of power and influence. They were also characterized by the development of strong player linkages between the Indigenous communities, companies, and to varying degrees, different levels of government as well as predominantly synergistic and reciprocal issue linkages.

In the Swedish Aitik case, company-community interactions involved continuous consultations spanning many decades and it appears that some collaborative initiatives have developed. Even so, the interactions were characterized as predominantly hierarchical. Both parties agreed that relationships have improved over time, but the RHC still perceives the interaction as unequal and lacking in terms of its ability to exert substantial influence (Poelzer et al., 2023). The interactions between the Swedish state authorities and the company were formally of a hierarchical nature, but involved a high degree of dialog and trust. By comparison, the formal institutions guiding the company’s interactions with the RHCs were weak. Issue linkages between the Gällivare RHC, which is struggling to maintain reindeer herding in the vicinity of the expanding Aitik mine, and the company were of an antagonistic and competitive nature. The company and the community had different experiences of the quality of their interactions, and player linkages with other actors in this mining dependent local community were weak (Poelzer et al., 2023).

The Prosperity and Kallak/Gállok cases (see MacPhail et al., 2022), involved open conflicts and very little company – community interaction occurred. In the Kallak/Gállok case, both Indigenous actors and the corporate project proponent assessed the quality of interaction as poor. The Indigenous communities involved in both cases deemed the assessment processes illegitimate due to a lack of meaningful consultation and biases in the way the assessment processes were conducted. In both cases, dialog broke down early and was hampered by perceived power imbalances, a lack of capacity on the part of Indigenous communities to participate effectively and overwhelmingly antagonistic and competitive issue linkages. These two cases also followed similar paths as different state authorities reached different conclusions about the mine’s impact and its compatibility with Indigenous livelihoods and rights (Wilson and Allard, 2023; MacPhail et al., 2022). These inconsistencies contributed to the delegitimization of hierarchically organized interactions with the state authorities: Indigenous and corporate actors tended to value their interaction with state actors that shared, and supported, their own perspectives, but distrusted state actors that went against them. Hence, player linkages between the Indigenous communities, the corporate actors, and the authorities supporting the mine projects, were weak in both cases. Instead, the Indigenous communities developed coalitions with human rights- and environmental NGOs.

The Prosperity case illustrated that a “state duty to consult” is, in itself, not a blueprint for high quality interactions and its practical application is not straightforward. Hence, the duty to consult is a procedural obligation upon the state, but the outcome of consultations is not given, and may vary depending on who is implementing it. However, compared to Sweden, the general Canadian institutional context provided the TNG with greater scope for legal redress in which they could challenge and change the course of interactions between the community, state and corporate actors (Wilson and Allard, 2023; MacPhail et al., 2022).

Returning to RQ 2, our analysis suggests that high quality interaction in the McArthur/Key Lake and Diavik cases was a product of a mix of different modes of interaction: well-functioning hierarchical-, co-, and self-governance models supported and reinforced each other. These arrangements offered the Indigenous communities opportunities to participate in processes which could provide synergistic and reciprocal issue and strong player linkages. Hence, mining negotiations in these cases could help raise, or reconcile concerns about Indigenous sovereignty. The quality of interaction scores lower in the other cases in which the modes of interactions were predominantly hierarchical (Prosperity and Kallak/Gállok), or hierarchical with elements of collaboration and self-governance (Aitik). These cases were characterized by antagonistic and competitive issue and weak player linkages, predominantly low-quality interaction which did not offer the Indigenous communities opportunities to realize long-term objectives as defined by them. Consequently, opposition and collaboration with NGOs seeking to protect the environment and Indigenous rights, proved a more attractive avenue.

### Outcomes and Governance Performance

The third research question sought to explain how the cases vary in terms of outcomes (opposition, acceptance, support) and performance. As shown in Table 3, the range of outcomes differed between Canada and Sweden. While mining related opposition and conflicts occurred in both countries, regardless of differences in institutions and company-community engagement practices, mutually appreciated collaboration and broad Indigenous support were only documented in some of the Canadian cases.

In three of the cases (McArthur River/Key Lake, Diavik and Aitik), the projects were given the necessary formal approvals, the mines are, or were operational, and the relationships between the mining companies and Indigenous communities resulted in formal agreements (Poelzer et al., 2023; Jackson et al., 2023; Poelzer, 2023). In the McArthur River/Key Lake and Diavik cases, the agreements between the respective mining companies and Indigenous communities were perceived as beneficial for both parties, and innovative collaborative practices and business partnerships evolved. No significant opposition was documented (Poelzer et al., 2023; Jackson et al., 2023). The interactions between the company and Gällivare Sámi RHC in the Aitik case also resulted in private agreements between the RHC and the mining company, and new modes of cooperation are currently being tested. However, these arrangements were not considered mutually satisfactory by all parties, and the RHC never gave their explicit consent to establish, or expand, the mines (Poelzer, 2023). All of these projects have resulted in operational mines but illustrated a range of outcomes: from acceptance and support in the Diavik and McArthur River/Key Lake cases; acceptance by compliance (in the absence of other alternatives) in the Aitik case; and opposition towards an expansion in mining activity involving a new mine in the Aitik/Liikavaara case.

In two of the cases, Kallak/Gállok and Prosperity, the mines were opposed by the Indigenous communities. In the Prosperity case, the project did not receive formal approval, an outcome that reflected the Indigenous community’s position. In the Kallak/Gállok case, the application was still under review at the time of investigation (McPhail et al., 2022). However, in March 2022, the Swedish Government approved the companies mining permit application with reference to economic benefits (Swedish Government 2022; Wilson and Allard, 2023), and the project can now proceed to an environmental assessment. While the TNG (Prosperity) has stated that it is not against mining as such (MacPhail et al., 2022), the position of the Swedish Sámi Parliament and Sámi RHCs is that no additional mines should be developed in Sápmi as long as Sámi rights are not properly recognized and protected in relevant legislation (Sámi Parliament, 2014).

The actors’ overall satisfaction with the performance of the mineral governance systems varied across the cases. The actors in the Canadian cases, where participation and collaboration between Indigenous communities and the mining companies had worked well (McArthur/Key Lake and Diavik), seemed to trust the governance system and appreciate its capacity to address their concerns (Poelzer et al., 2023; Jackson et al., 2023). In the other cases, the actors’ assessments of the governance systems, or different parts of them, seemed to reflect their interests in the outcomes. Generally, there were more concerns among Indigenous as well as corporate actors regarding the governance system, particularly the permitting process, in the Swedish cases (Mac Phail et al., 2022; Poelzer, 2023). The Sámi actors were deeply dissatisfied with the ability or willingness of the Swedish governing system to address their concerns about Sámi rights (Sámi Parliament, 2014).

In response to RQ3, we can conclude that outcomes vary, and that opposition, acceptance or support, also differ between the Canadian cases. Hence, general institutional factors such as the constitutional recognition of Indigenous rights, opportunities for litigation and the existence of a state duty to consult do not in themselves explain particular outcomes. Observed differences related to the provincial governments’ approaches and institutions (e.g., MSLAs and co-management boards), and the Indigenous communities’ and companies’ goals, strategies and practices are more likely to explain different outcomes in the Canadian cases. The Swedish cases generally displayed more opposition and less variation in terms of outcomes. Our comparative analysis illuminates important institutional differences which can help explain the broader range of outcomes in the Canadian cases. The governing systems in the Canadian cases offered the affected Indigenous community a broader set of tools to oppose and halt unwanted projects (e.g., Prosperity). However, they also provided instruments that helped corporate and Indigenous communities to respond in ways that generated broad Indigenous community support (e.g., McArthur/Key Lake and Diavik).

## Concluding Discussion

In the following section, we analyze and discuss our overarching research question: How do system properties and the type and quality of governing interactions interact to shape governability and produce different governing outcomes? Several important differences between the properties of the Canadian and Swedish governance systems, and the nature of observed governance interactions, affect governance challenges and help explain the variety of outcomes.

*First*, geography and the location of mines matter. Prosperity and Kallak/Gállok and Aitik involved mines in controversial locations of cultural, spiritual or economic importance to local Indigenous communities, and the Sámi RHCs’ overarching vision to maintain traditional reindeer herding added complexity to an already demanding governance task. However, the relationship between location/land use and Indigenous opposition is not straightforward. Under certain conditions, land use conflicts can be made more governable by proactive state and corporate governance measures. For example, in the McArthur/Key Lake case, the governing system responded to concerns by proposing MSLAs, and land use conflicts were addressed through collaboration and partnerships.

*Second*, there are important institutional differences between the cases, but more significantly between the countries that can help explain differences in governability and outcomes. In the Canadian cases where collaboration and partnerships were developed, the governing systems provided institutional instruments that helped corporate and Indigenous communities to respond in ways that enabled Indigenous community support. Governability was enhanced. Similar institutions were lacking, or were weaker, in the Swedish context. Institutional factors also appear to have affected governability and outcomes in the Prosperity and (ongoing) Kallak/Gállok cases. In the Prosperity case, stronger constitutional rights, a state duty to consult, more opportunity to litigate, and a more independent and inclusive federal EA assessment offered the affected Indigenous community a broader set of tools to defend their rights, culture and aspirations. In this sense, “societal concerns”, a key aspect of governability (Jentoft and Chuenpagdee, 2015a), could be addressed and the conflict was brought to closure by the Supreme Court of Canada (MacPhail et al., 2022). The Swedish governing system has so far proved incapable of handling the Kallok/Gállok project in ways that address societal concerns, handle conflicts in a manner that is perceived as fair and legitimate, and respect actors’ expectations on reasonable lead times and input of resources (MacPhail et al., 2022).

*Third*, corporate goals and strategies affect governability and outcomes. A number of variables explain the observed differences between the Swedish and Canadian cases: the space for “private” solutions (i.e., company-community “self- or co-governance”) was narrower in Sweden since most issues are handled as parts of the hierarchically organized state led permit process; the scope of the corporate practices and agreements applied in the Aitik case was much more limited compared to those in the McArthur/Key Lake and Diavik cases; and the corporate practices applied in the Swedish cases developed in the absence of the enabling governing measures that characterized the Canadian collaborative cases. In the latter, progressive corporate strategies in combination with an active and enabling state governing system promoted high quality governing interactions and broadly supported outcomes. By comparison, the Swedish context illustrated a kind of self-governance between two unequal partners in the absence of guidance, steering and control. In the Aitik case, the interactions were primarily hierarchical in the sense that power, resources and decision-making capacity were unevenly distributed between the companies and the Sámi RHC. This reflects the generally weaker position of Indigenous people in Sweden compared to Canada and the treatment of Sámi reindeer herding as a *public interest*. The conflict cases in both countries illustrated how inadequate, inappropriate, or insufficient, corporate engagement strategies may prevent the development of trust. However, they also demonstrated how particular systems characteristics (e.g., incompatible corporate and community visions and strategies) may constitute insurmountable governance hurdles for the realization of corporate visions to collaborate and co-exist with Indigenous communities.

*Fourth,* Indigenous communities’ goals, strategies and practices are properties of the system-to-be-governed and they are most clearly reflected in the interactions and outcomes that occurred in the cases. Analyzing issue and player linkages between the various actors revealed important differences which can help explain strategies, governing interactions and outcomes. According to Ali (2009), antagonistic and competitive issue linkages due to land use competition and clashing Indigenous and corporate values can aggravate governability and promote opposition. This was observed in the Prosperity and Kallak/Gállok cases, where player linkage with environmental and human rights NGOs grew stronger than with the corporate project proponents and this contributed to confrontation. In the cases where high quality governing interactions and broadly supported projects were documented, issue linkages were, or had become, predominantly synergistic and reciprocal. In these cases, critical issues were resolved through negotiation and processes that simultaneously strengthened Indigenous communities’ sense of respect, sovereignty and control. Hence, player linkage between the Indigenous communities, the companies and other governing actors grew strong, and collaboration and support developed. Accordingly, completely synergistic and reciprocal issue linkages at the outset of the projects did not seem to be a condition for the development of high-quality governing interactions, collaboration and Indigenous support. However, the possibility to develop synergistic and reciprocal issues linkages, i.e., compatible or mutually supportive corporate and community goals and development visions, appeared to be key to ensuring the governability of the system.

So, what do these differences mean in terms of governability and the performance of the governing systems? A key issue refers to the degree to which the governing system has the capacity to handle the characteristics of the system-to-be-governed and address societal concerns (Jentoft and Chuenpagdee, 2015a). Governability also depends on the ability of a governing system to deliver on the challenges that the system-to-be-governed raises (Kooiman and Bavinck, 2013). As our case studies illustrate, however, every situation is not equally governable, and the governing systems in the Swedish and Canadian cases responded to a variety of social concerns, but in different ways. None of these cases display a perfect fit or response. Nevertheless, we can observe that the governing systems in the Canadian cases offer Indigenous communities a broader set of tools to defend their rights, culture and aspirations, while actively promoting and supporting collaboration and partnerships where possible and appropriate. This did not always prevent intractable conflict, but it did promote a broader range of outcomes which, compared to the Swedish cases, appear to be more aligned with Indigenous community preferences. And, as evident in the McArthur/Key Lake and Diavik cases, this has not excluded mining. Consequently, the analysis suggests there are measures that can be taken by the Swedish Government to improve the governability of mining related issues, by developing alternative, and more effective, avenues to recognize, and protect, Sámi rights and culture, to broaden the scope and increase the legitimacy and transparency of the EIAs, to raise the quality of interaction and consultation, and to develop tools to actively stimulate and support collaboration and partnerships on equal terms.

Another key aspect of governability is how the governing system and the system-to-be-governed interact and, in particular, the modes and quality of interaction. The modes of interactions in the cases in which the quality of interaction scored low, were predominantly hierarchical, or at best hierarchical with elements of collaboration and elements of self-governance. The Aitik case illustrates how largely self-governed^7^ company-community activities, under unequal power relations and in the absence of supporting institutions, resulted in low quality company-community interactions and lack of trust – although private agreements were made. Interaction in hierarchical and co-governance modes, in contrast, typically take place within a formal mechanism where the division of labor, roles, rules, and responsibilities are clarified (Jentoft and Chuenpagdee, 2015a). The McArthur/Key Lake and Diavik cases involved a mix of self-governance, various sorts of co-governance and supporting hierarchical modes of governance, and illustrated how a healthy mix of modes can reinforce each other and promote high quality interactions. Consequently, and in line with interactive governance theory, this study shows that good quality interactions are necessary in all governing modes to promote governability. Jentoft and Chuenpagdee (2015b) particularly stress the importance of co-governance as a middle-of-the-road approach which engages the governing system, and the system-to-be-governed, in “bridging” and trust-building co-governance activities which are subject to negotiation, collaboration, and contextualization. The findings suggest that there are several instruments and approaches applied by Canadian governments and companies (e.g., MSLAs, co-management boards and a variety of agreements) that could be used by the Swedish government and corporate actors as models for promoting a higher degree of co-governance in the Swedish mining sector.

However, from the perspective of Indigenous communities, all governing modes are not equally attractive. As shown in our cases, the strategies chosen offered the Indigenous communities opportunities to participate in processes which could provide synergistic and reciprocal issue- and strong player linkages. While mining negotiations in the case of McArthur/Key Lake and Diavik helped forward, or reconcile, concerns about Indigenous sovereignty, opposition and collaboration with NGOs seeking to protect the environment and Indigenous rights proved more attractive avenues in the Prosperity and Kallak/Gállok cases. In line with Ali (2009), our study suggests that Indigenous community responses to mining must be understood within a larger framework of Indigenous self-determination. Accordingly, a hierarchical mode of governance is sometimes preferred over more devolved forms of decision making as hierarchical governance can encourage government-to-government interactions and the recognition of Indigenous sovereignty. According to interactive governance theory (Jentoft and Chuenpagdee, 2015a), revolts/resistance occur because of dissonance between the system-to-be-governed and the governing system; revolts are a way to communicate that dissonance. However, fully understanding Indigenous communities’ interactions and responses requires moving beyond an analysis of the fit between the system-to-be-governed, the governing system and modes of interaction. Indigenous communities’ own assessments of their opportunities to achieve their long-term objectives using alternative governing modes and types of interactions must be the center of attention, and the inclusion of Ali’s (2009) concepts about issue and player linkage into the interactive governance framework helped to illuminate this critical point.

According to Jentoft and Chuenpagdee (2015b), interactive governance must be true to principles of democracy, and both functional and normative aspects of governability should be emphasized (Kooiman, 2003; Kooiman et al., 2005; Bavinck et al., 2013). The normative aspect is about what goals and ethical choices are appropriate. While a complete assessment of the normative aspects of these cases is beyond the scope of this study, our analysis shows that normative governance issues are generally less discussed in the Swedish context. One way for governments to address the normative aspect of their engagement with Indigenous people, i.e., what is good, bad and desirable, is to embrace the principle of reconciliation. While the Government of Canada has actively promoted and engaged in a reconciliation process, similar state-led processes have not yet materialized in Sweden. A governing system that does not live up to prescribed normative standards is vulnerable, as it may not receive the legitimacy and support it needs to be sustainable (Jentoft and Chuenpagdee, 2015a). Also, it may not be true to principles of democracy and human rights. This study suggests that the normative aspect of governability associated with Indigenous use of natural resources warrants further attention.

### Supplementary Information

Appendix 1: Legal characteristics

## Footnotes

1 Vinnova funded project “What’s in a Social License to Mine? Indigenous, Industry and Government Best Practices for Social Innovation” (project No. 2017-02226), which is part of the national Swedish Strategic Innovation Program STRIM, a collaborative effort by Vinnova, Formas and the Swedish Energy Agency.

2 On an isolated 20 km^2^ island in Lac de Gras (Poelzer et al., 2023).

3 The *Constitution Act, 1982*, s. 35(2).

4 The *Constitution Act, 1982*, s. 35(1).

5 *Tsilhqot’in Nation v. British Columbia* [2014] SCC 44.

6 https://www.nrcan.gc.ca/sites/www.nrcan.gc.ca/files/mineralsmetals/files/pdf/rmd-rrm/Mine%20Surface%20Lease%20Agreement.pdf. (Accessed September 16, 2022).

7 Self-governance in the understanding of Jentoft and Chuenpagdee (2015a), i.e., referring to systems, or parts of systems, that have the ability to govern themselves without much external interference or support.
