# Supplementary material for: To Approve or not to Approve? A Comparative Analysis of State-Company-Indigenous Community Interactions in Mining in Canada and Sweden
Source: Environ Manage. 2024 Mar 6;73(5):946–61. doi: 10.1007/s00267-024-01949-7 (PMC11023974; doi:10.1007/s00267-024-01949-7)
Supplement: Supplementary file 1 — Appendix 1: Legal characteristics [file 267_2024_1949_MOESM1_ESM.docx]

Appendix 1. Legal characteristics

|  | Canada | Sweden |
| --- | --- | --- |
| System of government | Federal system. Power is constitutionally divided between the federal government and provincial governments; Canada’s northern territories are not part of this constitutional division of powers but, in recent years have acquired province-like powers through a process known as devolution.^[[1]](#footnote-1)^ | Unitary system.^[[2]](#footnote-2)^ |
| Division of powers | Federal level: Oversees matters that concern all of Canada, such as national defense, criminal law and fisheries.^^[[3]](#footnote-3)^^ Under section 91(24) of the *Constitution Act 1867*, the federal Parliament has legislative authority over ‘Indians, and Lands reserved for the Indians’, encompassing all three Indigenous peoples in Canada.^^[[4]](#footnote-4)^^  Provinces: Provincial governments also need to consider Indigenous rights when making decisions about resource development, and they have exclusive powers over property-, civil rights and generally all matters of a local or private nature in each province.^^[[5]](#footnote-5)^^ The governance on non-renewable natural- and forest resources is subject to exclusive provincial power.^^[[6]](#footnote-6)^^  Where there is a direct conflict between provincial and federal legislation, the doctrine of paramountcy provides that the federal laws prevail to the extent of inconsistency. | Sweden, as a unitary system, does not share these complicating government features. However, the long mining permitting processes, involve several state agencies and can show enough complexity.^[[7]](#footnote-7)^ |
| Differences of the legal systems (functions in different ways)^[[8]](#footnote-8)^ | Canada applies **common law** (except for Quebec). This implies a broader focus on litigation before courts in Canada, and, thus, the importance of courts in creating law. The Supreme Court of Canada (SCC) is the final court of appeal and has decided numerous cases concerning the content and protection of Indigenous rights as well as resource extraction. | Sweden adheres to the **civil law-tradition**. In civil law-countries, like Sweden, state legislation (and their preparatory works) is the prime legal source, and the courts, although important, do not have quite the same status or autonomy. Sweden has few cases from the Supreme Courts addressing the recognition of Indigenous Sami rights. Sweden has three different Supreme Courts relevant for this area of law. |
| Indigenous self-governance | In Canada there exist multitude of ways to support Indigenous communities’ self-governance. | In Sweden, the establishment of the Sami parliament in 1993 represents a first step towards a minimum of self-governance for the Sami, but the autonomy only concerns certain cultural and linguistic matters addressed in legislation.^[[9]](#footnote-9)^ |

1. Watts, R. L. (2008). Comparing Federal Systems (Montreal and Kingston: McGill-Queen’s University Press); Alcantara, C. 2013. ”Preferences, perceptions and veto players: explaining devolution negotiation outcomes in the Canadian territorial north.’ Polar Record, 49(2), 167-179. [↑](#footnote-ref-1)
2. See further Carlson, L., (2019), The Fundamentals of Swedish Law (Studentlitteratur). [↑](#footnote-ref-2)
3. See *Constitution Act 1867*, s. 91. Part VI of the Act allocates powers between the two levels of government: the exclusive legislative powers of the federal parliament are classified in subjects. Matters that are not listed in s. 91 can still be a matter of legislative interest of the federal parliament if the matter relates to ‘the Peace, Order, and good Government of Canada’, see the beginning of s. 91. [↑](#footnote-ref-3)
4. See also *Daniels v. Canada (Indian Affairs and Northern Development)* [2016] SCC 12. [↑](#footnote-ref-4)
5. *Constitution Act 1867*, s. 92(13). [↑](#footnote-ref-5)
6. *Constitution Act 1867* ss. 92, 92A. Municipalities, which are created under provincial laws, can make by-laws to deal with local matters, such as snow removal and parking. [↑](#footnote-ref-6)
7. Wilson, G. N. and Allard, C. (2022), “Institutional Determinants of Mining Projects in Canada and Sweden: Insights from the Prosperity and Kallak Cases”. Environmental Management. Special Section: Social license to mine: best practices. DOI https://doi.org/10.1007/s00267-022-01679-8. [↑](#footnote-ref-7)
8. Zweigert and Kötz, 2011. [↑](#footnote-ref-8)
9. Mörkenstam et al, 2016. [↑](#footnote-ref-9)
